# *Staphylococcus pseudintermedius*’s PBP4 Is Directly Associated with the Dissociated Oxacillin and Cefoxitin Phenotype

**DOI:** 10.3390/antibiotics10111299

**Published:** 2021-10-26

**Authors:** Paula Gagetti, Roberto R. Rosato, Adriana E. Rosato

**Affiliations:** 1 Servicio Antimicrobianos, INEI-ANLIS, Buenos Aires 1281, Argentina; pgagetti@anlis.gob.ar; 2 Houston Methodist Cancer Center, Houston Methodist Research Institute, Houston, TX 77030, USA; rrrosato@houstonmethodist.org; 3 Department of Pathology and Molecular Microbiology Diagnostics-Research, Riverside University Health System, Moreno Valley, CA 92555, USA; 4 School of Medicine, University of California Riverside, 900 University Avenue Riverside, Riverside, CA 92521, USA

**Keywords:** *S. pseudintermedius*, MRSP, Penicillin-Binding Proteins (PBPs), antimicrobial resistance

## Abstract

*Staphylococcus pseudintermedius* is an important pathogen responsible for infections in dogs and in humans. The emergence and dissemination of methicillin-resistant *S. pseudintermedius* (MRSP) and the multidrug resistance frequently seen in this species make difficult the treatment of these pathogens. The cefoxitin disk is widely used as a marker of methicillin resistance mediated by the *mecA* gene in *Staphylococcus aureus* and other staphylococcal species; however, it is not useful to detect β-lactam resistance of MRSP in clinical microbiology laboratories. The purpose of this study was to elucidate the molecular bases of the dissociated phenotype between oxacillin and cefoxitin antibiotics. By using a combinatorial approach that included the Penicillin-Binding Proteins’ (PBP) profile, their affinity for different β-lactam antibiotics and the analyses of PBPs’ sequence, we provide evidence that PBP4 showed still affinity for its target cefoxitin, impairing its phenotypic resistant detection in MRSP. Together, these findings provide evidence that *S. pseudintermedius* PBP4 is directly associated with the dissociated oxacillin and cefoxitin phenotype.

## 1. Introduction

*Staphylococcus pseudintermedius* (SP) is an important pathogen in dogs and cats and is sporadically associated with human infections. Over the past decade, Methicillin-resistant *S. pseudintermedius* (MRSP) has emerged worldwide [1]. In *S. aureus,* mechanisms of β-lactam resistance are linked to Penicillin-Binding Proteins (PBPs), critical components of the cell wall synthesis machinery in bacteria [2]. These membrane-associated proteins are broadly classified as low-molecular-mass (LMM), including PBPs that are monofunctional D,D-carboxypeptidase enzymes, or multimodular high-molecular-mass (HMM) PBPs, with multiple functional roles [3]. Although, PBPs are well characterized in *S. aureus*, in the SP species these proteins have not been fully investigated. 

Four native PBPs have been described in *S. aureus* including PBP1, a class B transpeptidase related to cell division [4], and PBP2, a dual-function class A enzyme with both transglycosylase and transpeptidase domains [5]. The transpeptidase domain of PBP2 is essential for the growth and viability of methicillin-susceptible strains (MSSA), a function that in methicillin-resistant *S. aureus* (MRSA) strains is replaced by PBP2A [6]. In fact, growth of MRSA in the presence of high concentrations of β-lactam antibiotics requires PBP2A and the transglycosylase domain of the native PBP2 [7]. PBP3 on the other hand is a class B HMM PBP with transpeptidase activity involved in septum formation and cell separation, while PBP4 is the only class B LMM bifunctional PBP in *S. aureus* and is involved in the secondary cross-linking of peptidoglycan [8]. However, PBP4 is not essential for cell growth under laboratory conditions, since mutants of *S. aureus* defective in PBP4 are still viable [3,9]. 

Similar to *S. aureus*, methicillin resistance in *S. pseudintermedius* is mediated by PBP2A, an additional PBP encoded by the *mecA* gene located in a mobile element of the bacterial chromosome called SCC*mec* (staphylococcal chromosomal cassette) [10]. Regulation of *mecA* takes place through *mecI*-*mecR1* and *blaI*-*blaR1* regulators. Other genes have also been shown to profoundly influence the resistance phenotype including multiple chromosomal factors such as the fem and aux factors, genes shown to play a role in peptidoglycan metabolism, revealing a complex relationship between cell wall metabolism and methicillin resistance [11,12]. 

Cefoxitin (FOX), a β-lactam antibiotic used as a surrogate marker of the *mecA* gene in *S. aureus* and other staphylococcal species, is a strong inducer of the *mecA* operon that is currently utilized to detect methicillin resistance. However, FOX it is not an accurate method of screening for methicillin resistance in *S. pseudintermedius* [13,14]. Given this limitation, in 2016 the Clinical and Laboratory Standard Institute (CLSI) recommended the use of the oxacillin (OXA) disk instead of FOX for the detection of methicillin resistance in *S. pseudintermedius*; a similar recommendation was recently issued by the European Committee on Antimicrobial Susceptibility Testing (EUCAST) [15]. However, the mechanistic bases linked to FOX failure in detecting methicillin resistance in MRSP is still unknown. The main goal of this study was to elucidate the molecular bases of the dissociated phenotype between OXA and FOX antibiotics in MRSP. By performing an array of combinatorial approaches including PBP-β-lactam affinity and genomic PBPs’ sequence analyses, we demonstrated that *S. pseudintermedius* PBP4 is directly associated with the dissociated FOX/OXA phenotype. 

## 2. Results

### 2.1. Binding to Major PBPs in S. pseudintermedius

**Figure 1:**
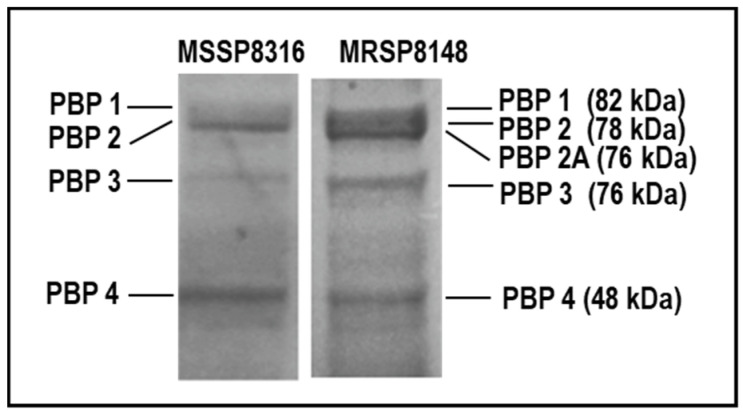
SDS-PAGE showing PBPs’ profiles of membrane proteins (100 µg) of methicillin-susceptible MSSP8316 (left panel, OXA MIC 0.12 µg/mL) and methicillin-resistant MRSP8148 (right panel, OXA MIC > 256 µg/mL) *S. pseudintermedius* isolates. Labeling of PBPs was performed as described in Methods.

The general profiles and identities of PBPs were examined in MSSP8316 and MRSP8148 strains. Strains MSSP8316 and MRSP8148 were chosen as representative of β-lactam-susceptible and -resistant *S. pseudintermedius*, respectively, for this study. The membrane abundance and activity of PBPs were assessed by fluorescent penicillin derivative Bocillin FL labelling. Membrane fractions extracted from both strains were incubated with bocillin and visualized by SDS-PAGE. All four native PBPs were distinctly identified in membranes’ samples (100 µg) from the MSSP8316 strain (Figure 1). In the absence of β-lactam exposure, bocillin affinity in MSSP8316 displayed a protein migrating at 82 kDa, corresponding to PBP1 as per its deduced amino acid sequence, and a nearby protein at 78 kDa, namely, PBP2. PBP3 and PBP4 were observed as proteins migrating at around 76 kDa and 48 kDa, respectively. Importantly, PBP4, the only low-molecular-mass PBP, was also found in *S. pseudintermedius*. A similar PBP1-to-4 profile appeared in membrane extracts of MRSP8148 with an additional protein migrating at 76 kDa, corresponding to PBP2A which, as seen in MRSA strains, appears very close to PBP2. 

These findings suggest that *S. pseudintermedius* expresses four native PBPs (1–4) in both MSSP8316 and MRSP8148 strains, with the presence in the latter of PBP2A, as similarly described in MRSA strains. 

### 2.2. PBP4 Is Linked to the Dissociated Resistant Phenotype between Oxacillin and Cefoxitin in S. pseudintermedius

**Figure 2:**
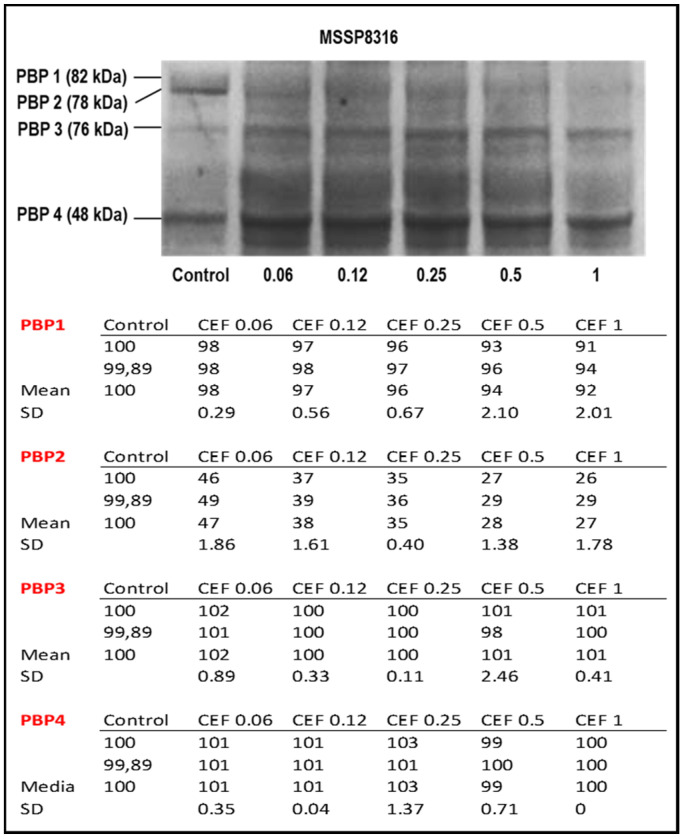
Upper panel: Affinities of MSSP8316 PBPs for cephalexin were determined by Bocillin-FL binding assay of membrane proteins of the corresponding MSSP8316 isolate preincubated with increasing concentrations of cephalexin (µg/mL); control: without preincubation. Lower panel: relative integrated density of the Bocillin-FL binding PBPs’ bands representative of two independent experiments performed by ImageJ. Values are expressed as mean and SD.

To compare the ability of different β-lactams in their binding ability to *S. pseudintermedius* PBPs, competition assays between β-lactams and bocillin in *S. pseudintermedius* were performed. Membrane extracts of MSSP8316 strain were incubated with increasing concentrations of cephalexin (0.06 µg/mL to 1 µg/mL) in the presence of labelled bocillin followed by SDS-PAGE detection. As shown in Figure 2, cephalexin was found binding PBP2 with high affinity as reflected by the highest binding at CEF 1 µg/mL and by a reduced amount of bound bocillin. In contrast, no changes were observed in PPB1, PBP3, or PBP4, which remained completely bound to Bocillin-FL (upper panel). The relative integrated density of the Bocillin-FL binding PBPs’ bands representative of two independent experiments performed by ImageJ is shown in the right panel.

**Figure 3:**
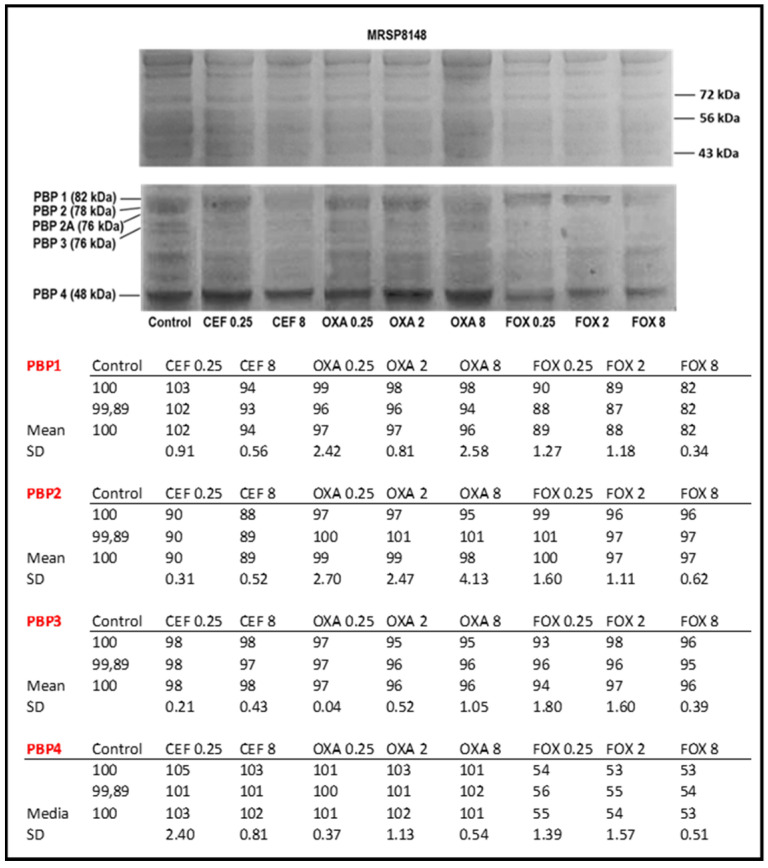
Upper panel: affinities of MRSP8148 PBPs’ isolate for three structurally different β-lactam antibiotics. Membrane proteins (100 µg) were prepared from MRSP8148 and analyzed by SDS-PAGE. Membrane extracts, except the one labeled Control, were pre-incubated with different concentrations (µg/mL) of cephalexin (CEF), oxacillin (OXA), and cefoxitin (FOX). Gels were developed by both Coomassie Blue staining (top gel) and Bocillin-FL binding assay (bottom gel). Lower panel: relative integrated density of the Bocillin-FL binding PBPs’ bands representative of two independent experiments performed by ImageJ. Values are expressed as mean and SD.

Competitive binding to Bocillin-FL was also performed on MRSP8148 strain membrane proteins preincubated with different concentrations of CEF (0.25 and 8 µg/mL), OXA (0.25, 2, and 8 µg/mL), and FOX (0.25, 2, and 8 µg/mL). While no changes were observed with the different β-lactams vs. the untreated control for CEF and OXA, increased binding of FOX to PBP4 at different concentrations was observed when compared to the untreated membrane protein control (Figure 3). Relative integrated density of the Bocillin-FL binding PBPs’ bands representative of two independent experiments was performed by ImageJ (Figure 3, lower panel). 

These findings may suggest (1) that PBP4 is still able to bind, at least in part, to its target FOX and (2) that oxacillin does no longer bind to its specific PBP1 and PBP2 targets. Furthermore, membranes preincubated with FOX showed less intensity of the bands corresponding to PBP1, PBP3, and PBP4 (Figure 3). Although the band corresponding to PBP4 was less intense in membranes preincubated with FOX, we did not observe a complete inhibition of PBP4.

The observed inhibition of PBP4 by cefoxitin in the competition assays together with the findings described above may suggest that PBP4 is directly related with the dissociated phenotype between oxacillin and cefoxitin characteristic of this species. In order to test this hypothesis, the amino acid sequence of PBP4 from the MRSP8150 strain was compared against all PBP4 sequences from *S. pseudintermedius* deposited at the GenBank database. A high degree of homology, i.e., more than 99% of amino acids’ identity, was found, indicating that PBP4 is a highly conserved protein in *S. pseudintermedius*. Similarly, amino acid sequence analysis of MRSP8150 PBP4 vs. its counterpart from *Staphylococcus schleiferi*, a species that has also shown a dissociated OXA-FOX phenotype, showed they shared more than 83% (352/425) identity. Furthermore, BLAST analysis with other *Staphylococcus* species excluding the *S. pseudintermedius* group (*Staphylococcus delphini*, *Staphylococcus cornubiensis,* and *Staphylococcus intermedius*) showed percentages of amino acid sequence identity greater than 96%. When compared against coagulase-positive or coagulase-variable staphylococci frequently isolated from animals, BLAST analysis of *S. pseudintermedius* PBP4 showed amino acid identity greater than 74% with *Staphylococcus lutrae*, *S. schleiferi*, *Staphylococcus fleuretti*, *Staphylococcus hyicus*, *Staphylococcus agnetis,* and *Staphylococcus chromogenes*. Thus, we concluded that PBP4 appears highly conserved across the species including *S. pseudintermedius,* expressing a dissociated OXA-FOX phenotype. 

### 2.3. Sequence Analysis of PBPs Encoding Genes in S. pseudintermedius

BLAST in silico analysis of the PBP1 sequence of *S. pseudintermedius* MRSP8150 strain (2223 base-pairs, 740 amino acids) showed 100% amino acid identity with *S. pseudintermedius* HKU10-03 reference strain (GenBank accession no. NC_014925.1) Furthermore, we found that PBP1 is the largest PBP of *S. pseudintermedius*, a class A bifunctional PBP of high molecular weight with transglycosylase and transpeptidase activity; in fact, PBP1 appears larger than its analog protein PBP2 in *S. aureus*. 

**Figure 4:**
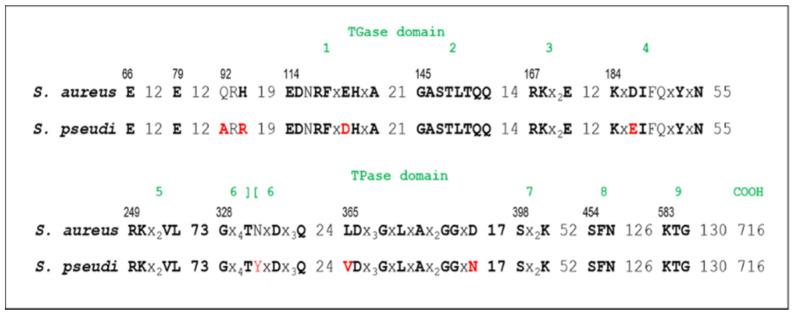
Comparison of amino acid sequence of *S. pseudintermedius* MRSP8150 PBP1 and the corresponding *S. aureus* N315 PBP2 homologue. Amino acid substitutions are indicated in red.

When compared to *S. aureus* N315 PBP2 (2184 bp and 727 amino acids), MRSP8150 PBP1 showed replacements in motifs usually conserved in class A PBPs that are responsible for their activity [5,16]. The amino acid changes corresponded to Q92A, H94R, and E120D in motif 1, D186E in motif 4, N334Y in motif 6, and L365V and D380N (Figure 4, highlighted in red). Moreover, these changes were found in both the MRSP isolates (MRSP8150) and *S. pseudintermedius* reference strain ED99 (GenBank accession no. NC_017568.1). 

In the case of *S. pseudintermedius* PBP2, it is composed of 2100 bp/699 amino acids and is analogous to *S. aureus* PBP1 (2232 bp/744 amino acids). Both proteins displayed a 70% amino acid identity (448/639), with *S. pseudintermedius* PBP2 not showing substitutions in any of the conserved motifs of class B PBPs responsible for its activity [17].

**Figure 5:**
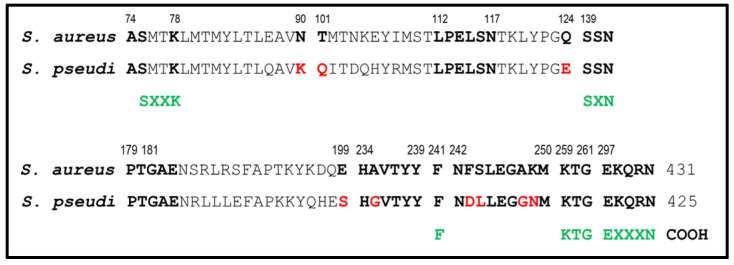
Comparison of PBP4 amino acid sequence of *S. pseudintermedius* MRSP8150 and *S. aureus* N315. Amino acid substitutions are indicated in red.

*S. pseudintermedius* PBP3 (2040 bp/679 amino acids) is analogous to *S. aureus* PBP3 (2076 bp/691 amino acids). BLAST analysis of PBP3 protein sequence showed 74% amino acid identity (466/632) compared to *S. aureus* N315 (GenBank accession no. BA000018), with no amino acid substitutions found in any of the conserved motifs previously described [17,18]. The corresponding *S. pseudintermedius* PBP4 (1278 bp/425 amino acids), analogous to *S. aureus* PBP4 (1296 bp/431 amino acids), is the only low-molecular-weight PBP in both species. MRSP8150 PBP4 showed 60% of amino acid identity compared with the *S. aureus* N315 PBP4 counterpart (Figure S1: Alignment of PBP4 amino acid sequence of MRSP8150 and *S. aureus* N315), with the presence of some substitutions in highly conserved regions (Figure 5).

*S. pseudintermedius* PBP2A has 2007 bp and 669 amino acids, similar to *S. aureus* PBP2A. Multiple alignments, i.e., seven out of 11 isolates including MRSP8148, MRSP8150, MRSP8468, MRSP8469, MRSP8472, MRSP8473, and MRSP8474, showed 100% nucleotide identity with *S. aureus* N315 PBP2A. In the remaining four isolates, (MRSP8151, MRSP8470, MRSP8471, and MRSP8510) we found a nucleotide change at position 675, resulting in an amino acid replacement at position 225 (S225R). These four isolates belonged to three different sequence types (STs) and displayed different levels of oxacillin resistance. 

In addition to PBPs, the *S. aureus* genome encodes four additional non-essential proteins with roles in peptidoglycan synthesis. They are two monofunctional transglycosylases, MGT and SgtA, and two auxiliary proteins, FmtA and FmtB [19]. By performing multiple alignment of *S. pseudintermedius* genomes and their amino acid sequences, we found that MGT has 265 amino acids and 768 bp, sharing 58% identity with *S. aureus* N315, while SgtA (299 amino acids and 900 bp) shares 53% identity with *S. aureus* N315. The MRSP FmtA transpeptidase has 407 amino acids, 1224 bp, and 48% identity to *S. aureus* N315 FmtA, with MSP FmtB being formed by 1566 amino acids and 4701 bp and showing between 39–43% identity with *S. aureus* N315 FmtB. 

In summary, this data analysis suggests that MRSP8150 possesses four native PBPs (PBP1 to PBP4) and an extra PBP2A, a protein that appears highly conserved in methicillin-resistant *Staphylococcus.* Furthermore, individual analysis of PBPs revealed that PBP1 is the largest one with bifunctional transglycosylase and transpeptidase domains, PBP2 and PBP3 with transpeptidase function and PBP4 with transglycosylase and carboxypeptidase activity. 

### 2.4. Analysis of Differential Gene Expression of MRSP 8150 under β-Lactam (Cephalexin) Exposure and Point Mutations in Specific Genes

To understand whether differences in gene expression could reflect the potential factors linking the response of MRSP strains to β-lactam antibiotics, we analyzed gene transcription levels by RNA-Seq comparing MRSP8150 grown in absence and presence of subinhibitory concentrations of cephalexin; a significant difference (>3-fold changes) in the expression of 1002 genes was observed (Table S1).

Decreased expression of *pbp4* and *fmtB* genes, the latter encoding the auxiliary transpeptidase FmtB responsible for the substitution of pentapeptides with pentaglycine, was observed (data not shown). Additionally, a decreased expression of the *pknB* gene, known as *aux2* encoding a serine-threonine kinase, was also detected. No differences were observed in the expression of the *fem* genes including *femA*, *femB,* and *femX*, which are normally highly conserved. However, some isolates showed mutations in the *femA* gene, resulting in amino acid changes to H49Q (MRSP8151, MRSP8470, MRSP8471, and MRSP8474), M113R (MRSP8510), A168T (MRSP8148, MRSP8468, MRSP8469, MRSP8473, and MRSP8510), and K420T (MRSP8510). The amino acid substitutions M113R and A168T were in a region that directly affects the binding and recognition capacity of Fem factors for the lipid chain. 

The *mprF* gene, which encodes the bifunctional lysyl-phosphatidyl-glycerol flippase/synthetase MprF, functionally involved in cell membrane synthesis, appeared downregulated in MRSP8150 while no changes in genes associated with peptidoglycan precursors’ synthesis, including *murA*, *glmS*, *lacG*, *lysA,* and *thrS*, were seen. In addition, a decreased expression of *glpQ*, a gene encoding the glycerophosphodiesterase GlpQ (which cleaves a broad variety of glycerol-3-phosphate headgroups of deacylated phospholipids), was also identified. Other genes downregulated were those involved in DNA metabolism including the gene that encodes the α subunit of DNA polymerase III; *radA* (DNA repair); *recG* and recQ (encoded RecG and RecQ recombinases, respectively); and *dinB* (encodes DNA polymerase IV). 

The expression level of genes under conditions of stringent stress induced by the addition of subinhibitory concentrations of cephalexin to the growth medium was also evaluated. Among these genes we observed decreased expression of *hpt* (which encodes hypoxanthine phosphoribosyltransferase), *gltB* (which encodes the α subunit of glutamate synthetase), *hemL* (which encodes glutamate-1-semialdehyde 2,1 aminomutase), *guaA* (which encodes GMP synthetase), *cysK* (which encodes cysteine synthetase A), and *rplN* and *rplP* (which encode ribosomal proteins L14 and L16, respectively). Additionally, the expression of some regulatory systems was affected including the decreased expression of *yycH* (which encodes the histidine kinase sensor, a signal transducer of the two-component regulatory system YycFG, related to biofilm formation and cell wall turnover) and *norA* (which encodes a Major Facilitator Superfamily efflux pump and is very important in antibiotic resistance). Additionally, the decreased expression of several phage-associated genes encoding phage capsid proteins, phage tail proteins, phage head morphogenesis proteins, head–tail adapter protein, phage portal proteins, and a phage antirepressor protein, were also observed, as well as three genes encoding pathogenicity islands. 

Altogether, these findings demonstrated that MRSP differential β-lactam resistance is manifested by changes in regulation, downregulation of PBP4, and downregulation of genes associated to the DNA metabolism.

## 3. Discussion

Since 2006, MRSP has emerged as a significant animal health problem in veterinary medicine [20]. It is well known that the FOX disk is not recommended for the detection of methicillin resistance in *S. pseudintermedius* [14,21,22,23]. However, the reason for the discrepancy with *S. aureus* phenotypic β-lactam-resistant detection and the precise molecular mechanism of the dissociation between OXA and FOX in MRSP remains to be elucidated. Moreover, the role and function of *S. pseudintermedius* PBPs, the primary target of β-lactam antibiotics, is scarce. Early studies by Canepari et al. showed the profile of the PBPs on five species of staphylococci of animal origin including the description of three PBPs in *S. intermedius* with molecular sizes of 85, 82, and 79 kDa [24]. Until 2005, when it was differentiated as a species, *S. pseudintermedius* was still identified as *S. intermedius* [25]. The present study expands our knowledge on PPBs in more contemporaneous MRSP clinical isolates obtained from infected dogs. 

MRSP encodes nine enzymes for peptidoglycan synthesis. One of them, PBP2A, was acquired from other species by a mobile genetic element [26]. The core eight enzymes are PBP1, a bifunctional transglycosylase and transpeptidase; PBP2, a monofunctional transpeptidase; PBP3, a transpeptidase; PBP4, a LMM transpeptidase; two monofunctional transglycosylases, MGT and SgtA; and two auxiliary transpeptidases (FmtA and FmtB).

Our findings show PBP1, a class A bifunctional transglycosylase and transpeptidase PBP, as the largest one in *S. pseudintermedius*, with a higher molecular weight than its *S. aureus* PBP2 analog. As shown in previous studies, the dicarboxylic amino acid residues E and D in motif 1 and E in motif 3 of class A PBPs are both essential components of the transglycosylase catalytic module site of *S. aureus* PBP2 and are highly conserved in class A PBPs of different bacterial species [16]. Our results demonstrated in *S. pseudintermedius* PBP1 the presence of seven amino acid substitutions in three motifs usually conserved and responsible for the activity of class A PBPs. Furthermore, our data indicated that PBP2 is a high-molecular-mass class B PBP with transpeptidase function, analogous to *S. aureus* PBP1. In addition, we showed that PBP3 is a class B high-molecular-mass PBP with transpeptidase function analogous to the *S. aureus* PBP3, while PBP4 was the only low-molecular-mass class B PBP, with transglycosylase and carboxypeptidase activity analogous to the *S. aureus* PBP4.

The structure of *S. aureus*’s PBP4 contains some well-characterized regions located at or near the active site, which catalyzes acyl exchange reactions that occur during cell wall turnover. The active site in the case of PBP4 is also formed by the arrangement of SXXK, SXN, and KTG motifs at the interface of an antiparallel β-sheet and the larger α-helical cluster of the N-terminal domain [3]. The SXXK motif is present in an α-helix, the SXN motif is in a loop, and the KTG motif is present in a β-strand. Along with these motifs, two loops incorporating P179 to E183 and L112 to N117 form the boundaries of the active site. In PBP4 as well as all class A β-lactamases, an additional motif, EXXXN present on the so-called Ω loop, is responsible for the high rates of diacylation. K78 and the coiled segments H234 to T239 and N242 to M250 are also essential to catalyze acylation and diacylation reactions [3]. Importantly, we found five amino acid substitutions locating to these regions in the PBP4 sequence of the isolates under study. In this sense, it would be plausible to speculate that these structural alterations may favor PBP4 to preserve in part its activity in the presence of cefoxitin in MRSP, as was shown by binding activity approaches. It is also possible that PBP4 is acting in concert with PBP2A in MRSP isolates, considering that similar interactions exist in MRSA between PBP2 transglycosylase and PBP2A transpeptidase activity, all together to sustain methicillin resistance. However, the evidence of a mutual interaction between PBP4 and PBP2A awaits experimental confirmation. 

To understand whether differences in gene expression could reflect the potential factors linking the response of MRSP strains to β-lactam antibiotics, MRSP was grown in the presence of subinhibitory concentrations of cephalexin, an antibiotic extensively used in MRSP dog infections. These analyses reflected a decreased expression of *pbp4*, *fmtB,* and *aux2* genes that are related with peptidoglycan synthesis and downregulation of the *mprF* gene involved in the cell membrane synthesis. Several genes involved in DNA metabolism and strict stress response were downregulated as well. In line with these observations, genes related to DNA metabolism and the strict stress response have been shown to cause an increase in PBP2A expression together with a phenotypic increase in β-lactam resistance levels [27,28].

The non-identification of a candidate gene responsible for the different levels of resistance to β-lactams shown by the MRSP isolates would indicate a multifactorial phenomenon, driven by the accumulation of mutations in multiple genes and factors that modify the expression of resistance.

Collectively, this study led to novel findings, suggesting that PBP4 is a key element in the dissociated phenotype between OXA and FOX. This claim is supported by the results of structural analysis and the affinity that FOX still exhibits by PBP4. These characteristics appear relevant to *S. pseudintermedius* and *S. schleiferi,* where this discrepant phenotype has been observed. Altogether, these results showed that all the native PBPs of *S. pseudintermedius* are closely related to the PBPs of staphylococcal species with a common evolutionary origin frequently isolated in animals. 

## 4. Materials and Methods

### 4.1. Bacterial Strains

In the present study 10 canine clinical MRSP isolated in Argentina from 2008 to 2011 were included alongside with one MRSP isolated from a human patient in 2017. These strains were previously characterized by antimicrobial resistance, SCC*mec* typing, pulsed-field gel electrophoresis (PFGE), multilocus sequence typing (MLST), and whole genome sequencing (WGS) [29,30]. Additionally, a methicillin-susceptible *S. pseudintermedius* strain (Oxacillin MIC 0.25 µg/mL) was used [31]. 

MRSP8148 and MRSP8150 had oxacillin MIC > 256 µg/mL. MRSP8148 was used by proteome analysis and MRSP8150 was used to perform sequences’ comparison and RNA-Seq analysis.

### 4.2. Total Protein Extracts

Overnight cultures of strains growing in Mueller–Hinton broth (MHB) at 37 °C with agitation were diluted (1/100) in the same medium and grown at 37 °C with agitation to an OD600 of 0.5. After centrifugation, the cell pellets were washed and resuspended in 500 μL TE buffer (10 mM Tris, pH 7.5, 1 mM EDTA). Bacterial cells were disrupted by adding 500 μL of acid-washed glass beads (100 to 200 μm; Sigma) in a FastPrep cell disrupter (MP Biomedicals, Santa Ana, CA, USA). The cell debris was separated from soluble protein extracts by centrifugation at 14,000 rpm (10 min at 4 °C). The supernatant was concentrated in Amicon 10,000-molecular-weight-cutoff centrifugal filters (Millipore Sigma, Burlington, MA, USA) to a final volume of 40 µL. Ponceau staining was used as protein loading control.

### 4.3. Membrane Extracts

Membrane proteins were isolated, as previously described [32]. Briefly, strains were grown in Triptic soy broth (TSB) until the mid-exponential phase and pellets were resuspended in 1/250 of the initial culture volume of buffer A (50 mM KPO4 buffer [pH 7.4], 10 mM MgCl2). Bacterial cells were disrupted by using a FastPrep cell disrupter (MP Biomedicals) after which the resulting lysate was centrifuged at 7000 rpm for 30 min at 4 °C. The supernatant was centrifuged at 80,000 rpm in a Beckman ultracentrifuge TL100 using a TLA 100.3 rotor for 1 h at 4 °C. The membrane pellet was washed with buffer B (50 mM KPO4 [pH 7.4], 10 mM MgCl2, 20% glycerol) and then resuspended in the same buffer. Total membrane proteins were quantified using a BCA kit assay (Thermo Fisher Scientific, Waltham, MA, USA), diluted to 10 mg/mL in buffer B, snap-frozen with liquid N2, and stored at −80 °C. 

### 4.4. Bocillin Labeling

Portions (100 µg) of membrane proteins were labeled with 100 µM bocillin-FL (Molecular Probes, Thermo Fisher Scientific, Waltham, MA, USA) for 10 min at 30 °C. The reaction was stopped by adding 5-fold-concentrated SDS-PAGE sample buffer (500 mM dithiothreitol, 10% SDS, 250 mM Tris-HCl [pH 6.8], 30% glycerol, 0.02% bromophenol blue). Labeled membrane proteins (20 µg) were separated on a 7.5% SDS-PAGE gel and detected using a 473-nm laser of a Fuji FLA-5100 reader. The quantification of the intensity of the fluorescent bands was performed using ImageJ software. These results are representative of two independent experiments.

### 4.5. In Silico Sequences’ Comparison Analysis

The PBP sequences of MRSP isolates were compared with those corresponding to reference strains of *S. aureus* and *S. pseudintermedius*. Accession numbers of PBPs’ sequences used in comparisons are shown in Table 1.

The nucleotide and predicted amino acid sequences were analyzed using NCBI BLAST. Available online: https://www.ncbi.nlm.nih.gov/blast/ (accessed on 22 October 2021).

Multiple alignment was performed by Cobalt. Available online: https://www.ncbi.nlm.nih.gov/tools/cobalt/cobalt.cgi?CMD=Web (accessed on 22 October 2021) and ClustalX, available online: http://www.clustal.org/clustal2/ (accessed on 22 October 2021).

### 4.6. RNA-Seq Expression Analysis

Overnight bacterial cultures grown in MH broth at 37 °C and shaken at 150 rpm were diluted 1:100 in MH and incubated until they reached an OD600 of ~0.5. RNAlater reagent (Qiagen, Inc., Valencia, CA, USA) was added to bacterial cell cultures to protect the cellular RNA. Total RNA extraction was extracted by using a RNeasy isolation kit (Qiagen), and the DNA was removed using a DNA-free DNA removal kit (Thermo Fisher Scientific). RNA concentrations were assessed by measuring absorbance at 260 and 280 nm using a NanoDrop 8000 (Thermo Fisher Scientific). For RNA-Seq analysis, RNA was prepared from cells collected during the exponential phase of growth of the strains MRSP8150, and MRSP8150 treated with CEF 4 µg/mL. The quality of the total RNA was assessed using RNA Nano chips (Agilent Technologies, Santa Clara, CA, USA) run with an Agilent 2100 Bioanalyzer and 2100 Expert software. The genome-wide transcript sequencing libraries were prepared according to the manufacturer’s recommendations (ScriptSeq; Epicentre BIOtechnologies, Madison, WI, USA) and sequenced on a MiSeq instrument (Illumina, San Diego, CA, USA). Differential gene expression was determined using Lasergene (v14) software (DNAStar, Madison, WI, USA); differences of >3-fold and *p* < 0.05 after applying Bonferroni correction were considered significant.

## 5. Conclusions

This study showed that PBP4 is a critical element of the dissociated phenotype between OXA and FOX. Structural analysis of the PBP4 of the isolates under study showed five amino acid substitutions in regions highly conserved of the PBP4. These substitutions could be related to the conserved binding activity of PBP4 in the presence of cefoxitin, as was demonstrated by binding activity approaches. Some of these amino acid replacements were also found in the PBP4 sequences of other staphylococcal species frequently isolated in animals, suggesting that the cefoxitin disk might not be helpful in detecting methicillin resistance in these species.

## Figures and Tables

**Table 1:** *S. aureus* and *S. pseudintermedius* PBPs’ accession numbers.

Protein	Accession Number
*S. aureus* N315 PBP1	BAB43267.1
*S. aureus* N315 PBP2	BAB42543.1
*S. aureus* N315 PBP3	BAB42644.1
*S. aureus* N315 PBP4	BAB41830.1
*S. aureus* N315 PBP2A	BAB41256.1
*S. aureus* N315 Mgt	WP_000830380.1
*S. aureus* N315 SgtA	WP_000184370.1
*S. aureus* N315 FmtA	WP_000671245.1
*S. aureus* N315 FmtB	WP_001048266.1
*S. pseudintermedius* ED99 PBP1	ADX76608.1
*S. pseudintermedius* ED99 PBP2	ADX76886.1
*S. pseudintermedius* ED99 PBP3	ADX76509.1
*S. pseudintermedius* ED99 PBP4	ADX77343.1
*S. pseudintermedius* ED99 Mgt	ADX76192.1
*S. pseudintermedius* ED99 SgtA	ADX76335.1
*S. pseudintermedius* ED99 FmtA	ADX77189.1
*S. pseudintermedius* ED99 FmtB	ADX75447.1

## Data Availability

RNA-Seq data corresponding to the differential gene expression of MRSP8150 strain treated and treated with subinhibitory concentrations of cephalexin is available at Sequence Read Archive (SRA; https://www.ncbi.nlm.nih.gov/sra (accessed on 22 October 2021)) under bioproject accession PRJNA770087.

## Supplementary Materials

The following are available online at https://www.mdpi.com/article/10.3390/antibiotics10111299/s1. Figure S1: Alignment of PBP4 amino acid sequence of MRSP8150 and *S. aureus* N315. Figure S2: Multiple alignment of PBP4 from MRSP8150 and PBP4 of species shared more than 74% of amino acid identity. *S. aureus* PBP4, which shares only 60% of amino acid identity, was included for illustrative purposes. Table S1: Differential gene expression of MRSP8150 untreated and -treated with subinhibitory concentrations of cephalexin.
