# Supplementary material for: Staphylococcus pseudintermedius’s PBP4 Is Directly Associated with the Dissociated Oxacillin and Cefoxitin Phenotype
Source: Antibiotics (Basel). 2021 Oct 26;10(11):1299. doi: 10.3390/antibiotics10111299 (PMC8614965; doi:10.3390/antibiotics10111299)
Supplement: Supplementary file 1 [file antibiotics-10-01299-s001.zip › antibiotics-1347781-supplementary figures S1 and S2.pdf]

Figure S1. Alignment of PBP4 amino acid sequence of MRSP8150 and S. aureus N315 (GenBank accession no. BA000018).

```
PBP4_SAU_N315    1 MKNLISIIIILCLTLSIMTPYAQATNSDVTPVQAANQYGYAGLSAAYEPTSAVNVSQTGQLLYQYNIDTKWNPASMTKLM 80
PBP4_MRSP8150    1 MKKIILSIVVVFFASTIITPFARAYTP--TPVELAQQSGYP-VTWAYQPEGMINISQTGQILYEFQSEQQWYPASMTKLM 77

PBP4_SAU_N315   81 TMYLTLEAVNKGQLSLDDTVTMTNKEYIMSTLPELSNTKLYPGQVWTIADLLQITVSNSSNAAALILAKKVSKNTSDFVD 160
PBP4_MRSP8150   78 TMYLTLQAVKEKKLSLNDTVQITDQHYRMSTLPELSNTKLYPGETYTVAELLQITVSASSNAAALILANQVSDSTSDFVD 157

PBP4_SAU_N315  161 LMNNKAKAIGMKNTHFVNPTGAENSRLRSFAPTKYKDQERTVTTARDYAILDLHVIKETPKILDFTKQLAPTTHAVTYYT 240
PBP4_MRSP8150  158 KMNDTAKSLGMTHTHYVNPTGAENRLLLEFAPKKYQHESSSTSSARDYAILAQHVVKDTPKILYFTKQIAPTQHGVTYYT 237

PBP4_SAU_N315  241 FNFSLEGAKMSLPGTDGLKTGSSDTANYNHTITTKRGKFRINQVIMGAGDYKNLGGEKQRNMMGNALMERSFDQYKYVKI 320
PBP4_MRSP8150  238 FNDLLEGGNMSLPGTDGLKTGSSDIADYNNSLTTKRGKFRIFHIIMGAGDYKHLGGEKQRNMMSASAINYTFSQYDYKKI 317

PBP4_SAU_N315  321 LSKGEQRINGKKYYVENDLYDVLPSDFSKKDYKLVVEDGKVHADYPREFINKDYGPPTVEVHQPIIQKANTVAKSMWEEH 400
PBP4_MRSP8150  318 VSKGKHKIDGKSYYVTEDLYDVVPKDMSKP-YHLVVKDSQVHLDYPRKFITKNDGPPTVKVENPLIYESKSVVVSSWHAF 396

PBP4_SAU_N315  401 PLFTIIGGACLVAGLALIVHMIINRLFRKRK 431
PBP4_MRSP8150  397 PILTSLAFLVIVLILAFILRRLLKLLFRK-- 425
```

Figure S2. Multiple alignment of PBP4 from MRSP8150 and PBP4 of species shared more than 74% of amino acid identity. *S. aureus* PBP4 which shares only 60% amino acid identity and was included for illustrative purposes.

```
S. pseudintermedius 1   --MKKIILSIVVVFFASTIITP--FARAYTPTPVELAQQSGYP-VTWAYQPEGMINISQTGQILYEFQSEQQWYPASMTK 75
S. delphini         1   --MKKIILSIVVVFFASTIITP--FARAYTPTPVELAQQSGYP-VTWAYQPEGMINISQTGQILYTFQSEQQWYPASMTK 75
S. cornubiensis     1   --MKKIILSIVVVFFASTIITP--FARAYTPTPVELAQQSGYP-VTWAYQPEGMINISQTGQILYDYQSEQQWYPASMTK 75
S. intermedius      1   --MKKIILSIVVVFFASTIITP--FARAYTPTPVELAQQSGYP-VTWAYQPEGVMNISQTGQILYDFQSEQQWYPASMTK 75
S. lutrae           1   --MKKLILSIVVVFFATTIITP--FARAYTPTPVELAQQSGYP-VTWPYQPEGMINISETGQILYNYHSNQQWYPASMTK 75
S. schleiferi       1   mqMKKIILSIVVVFFATTIITP--FARAYTPSPVELAQQSGYP-VTLPFQPEGVINISQTGQILYDYNSQKEWYPASMTK 77
S. coagulans        1   --MKKLILSIVVVFFATTIITP--FARAYTPSPVELAQQSGYP-VTLTFQPEGVINISQTGQILYDYNSQKEWYPASMTK 75
S. fleurettii       1   --MKKLILSIVAVFFATTIITP--FARAYTPSPVELAQQSGYP-VTLPFQPEGVINISQTGQILYDYNSQKEWYPASMTK 75
S. hyicus           1   --MKKVILSILVVFFATTIITP--FTKANTATPVDIANQYGYA-VGYGFQPEGLVNISETGQILYQFQDKQKWYPASMTK 75
S. agnetis          1   --MKKVILSILVVFFATTIITP--FTKANTATPVDIANQNGYG-VGYGFQPEGLVNISETGQILYQYQDKQKWYPASMTK 75
S. chromogenes      1   --MKKVILSILVVFFATTIITP--FTKANTVTPVDIANQYGYS-VGYGFQPEGLINISETGQILYQYQDQQKWYPASMTK 75
S. aureus           1   --MKRRFTMILVALLTIAIIAPktYASEYEPTPTEIARQYGYPnVTDAYQPEGSINVSQTGQILYDYQSNQKWYPASMTK 78

S. pseudintermedius 76  LMTMYLTLQAVKEKKLSLNDTVQITDQHYRMSTLPELSNTKLYPGETYTVAELLQITVSASSNAAALILANQVSDSTSDF 155
S. delphini         76  LMTMYLTLQAVKEKKLSLNDTVQITDQHYRMSTLPELSNTKLYPGETYTIAELLQITVSASSNAAALILANQVSDNTSDF 155
S. cornubiensis     76  LMTMYLTLQAVKEKKLSLNDTVQMTDQHYRMSTLPELSNTKLYPGETYTIAELLQITVSASSNAAALILANQVSDNTSDF 155
S. intermedius      76  LMTMYLTLKAVKEKKLSLNDTVQITDQHYRMSTLPELSNTKLYPGETYTIAELLQITVSASSNAAALILANQVSDNTSDF 155
S. lutrae           76  LMTMYLTLQAVKEKKLSLNDTVKITDQHYRMSTLPELSNTKLYPGEIYTIAELLQITVSASSNAAALILANQVSGNTSDF 155
S. schleiferi       78  LMTMYLTLQAVKEKKLSLNDTVKITPEHYRMSTLPELSNTKLFPGETYTIAELLQITVSASSNAAALILANQVSDNTSDF 157
S. coagulans        76  LMTMYLTLQAVKEKKLSLNDTVKITPEHYRMSTLPELSNTKLYPGETYTIAELLQITVSASSNAAALILADQVSDNTSDF 155
S. fleurettii       76  LMTMYLTLQAVKEKKLSLNDTVKITPEHYRMSTLPELSNTKLYPGETYTIAELLQITVSASSNAAALILANQVSDNTSDF 155
S. hyicus           76  LMTMYLTLKAVKSGTLNLNDTVKITNTHYRMSTLPELSNTKLYPGETYTIAELLQITVSASSNAASLILAQKVSGSTSQF 155
S. agnetis          76  LMTMYLTLKAVKSGDLNLNDTVKITDQHYRMSTLPELSNTKLYPGETYTIAELLQITVSASSNAASLILAQKVSGSTSKF 155
S. chromogenes      76  LMTMYLTLQAVKSGDLSLDDTIKITDEHYRMSTLPELSNTKLYPGETYTIAELLQITVSASSNAASLILAKKVSGSTSKF 155
S. aureus           79  LMTMYLTLEAINKGELSLNDKVHITDQHYRMSTLPELSNTKLYPGETYTISELLQITVSNSSNAAALILADEVSGNVNDF 158

S. pseudintermedius 156 VDKMNDTAKSLGMTHTHYVNPTGAENRLLLEFAPKKYQHESSSTSSARDYAILAQHVVKDTPKILYFTKQIAPTQHGVTY 235
S. delphini         156 VDKMNDTAKSLGMTHTHYVNPTGAENRLLLEFAPKKYQHESSSTSSARDYAILAQHVVKDTPKILYFTKQIAPTQHGVTY 235
S. cornubiensis     156 VDKMNDTAKSLGMTHTHYVNPTGAENRLLLEFAPKKYQHESSSTSSARDYAILAQHVVKDTPKILYFTKQIAPTQHGVTY 235
S. intermedius      156 VDKMNDTAKSLGMTHTHYVNPTGAENRLLLEFAPKKYQHESSSTSSARDYAILAQHVVKDTPKILHFTKQIAPTQHGVTY 235
S. lutrae           156 VDKMNETAKALGMTHTHYVNPTGARNRLLLEFAPEKYRNEASSTSTPRDYAILALHTVKDTPKILDFTKQIAPTQHGVTY 235
S. schleiferi       158 VDKMNQTAKSLGMNHTHYVNPTGAENRLLLEFVPKRYQNESSSTSTAHDYAILAQHTVQDTPKILHFTKQIAPTQHGVTY 237
S. coagulans        156 VDKMNQTAKSLGMNHTHYVNPTGAENRLLLEFVPKRYQNESSSTSTAHDYAILAQHTVQDTPKILHFTKQIAPTQHGVTY 235
S. fleurettii       156 VDKMNQTAKSLGMNHTHYVNPTGAENRLLLEFVPKRYQNESSSISTAHDYAILAQHTVQDTPKILHFTKQIAPTQHGVTY 235
S. hyicus           156 VDDMNQTAKSLGMTQTHYVNPTGAENFRLQEFVPEKYSHETSSISSPRDYAILAQHAVQDTPKILDFTKQIAPTQHGVTY 235
S. agnetis          156 VDDMNQTAKDLGMTQTHYVNPTGAENFRLQEFVPQKYKNETSSVSSPRDYAILAQHTVQDTPKILDFTKQIAPTQHGVTY 235
S. chromogenes      156 VDDMNQTAKELGMSKTHFVNPTGAENYRLQEFAPKKYKNEMSSVTSPHDYAILALHTVKDTPKILDFTKQIAPTQHGVTY 235
S. aureus           159 TDLMNKKAKALGMSNTHFVNPTGAENSQLKDFAPSKYKDQDNSTSTAKDFAILDHHVIKETPKILHFTNQLAPTQHGVTY 238

S. pseudintermedius 236 YTFNDLLEGGNMSLPGTDGLKTGSSDIADYNNSLTTKRGKFRIFHIIMGAGDYKHLGGEKQRNMMSASAINYTFSQYDYK 315
S. delphini         236 YTFNDLLEGGNMSLPGTDGLKTGSSDIADYNNSLTTKRGKFRIFHIIMGAGDYKHLGGEKQRNMMSASAINYTFSQYDYK 315
S. cornubiensis     236 YTFNDLLEGGNMSLPGTDGLKTGSSDIADYNNSLTTKRGKFRIFHIIMGAGDYKHLGGEKQRNMMSASAINYTFSQYDYK 315
S. intermedius      236 YTFNDLLEGGSMSLPGTDGLKTGSSDIADYNNSLTTKRGKFRIFHIIMGAGDYKHLGGEKQRNMMSASAINYTFSQYDYK 315
S. lutrae           236 YTFNDLLEGGNMSLPGTDGLKTGSSDIADYNNSLTTKRGAFRIFHIIMGAGDYRHLGGEKERNMMSASAINYSFAQYDYK 315
S. schleiferi       238 YTFNDLLEGGNMSLEGTDGLKTGSSDIADYNNSLTTKRGKFRIFHIIMGAGDYKHLGGEKQRNMMSASAINYSFALYDYK 317
S. coagulans        236 YTFNDLLEGGNMSLEGTDGLKTGSSDIADYNNSLTTKRGKFRIFHIIMGAGDYKHLGGEKQRNMMSASAINYSFALYDYK 315
S. fleurettii       236 YTFNDLLEGGNMSLEGTDGLKTGSSDIADYNNSLTTKRGKFRIFHIIMGAGDYKHLGGEKQRNMMSASAINYSFALYDYK 315
S. hyicus           236 YTFNDLLEGGDMSLPGTDGLKTGSSDLADYNNTLTTKRGKFRIFHVIMGAGDYKNLGGEKERNMMSASTINAAFNQYDYR 315
S. agnetis          236 YTFNDLLEGGNMSLQGTDGLKTGSSDLADYNNTLTTKRGKFRIFHVIMGAGDYKNLGGEKERNMMSASTINVAFYQYDYR 315
S. chromogenes      236 YTFNDLLEGGNMSLQGTDGLKTGSSDLADYNNSLTTKRGKFRIFHIIMGAGDYKNLGGEKERNMMSASTINAAFDQYDYK 315
S. aureus           239 YTFNHSLEGADMSLPGTDGLKTGSSDVADYNHTITTKRKQFRINQVIMGAGDYVSLGGEKQRNMMGNAMMNRSFDQYRYK 318
```

```
S. pseudintermedius 316 KIVSKGKHKIDGKSYYVTEDLYDVVPKDMSK-PYHLVVKDSQVHLDYPRKFITKNDGPPTVKVENPLIYESKSVVVSSWH 394
         S. delphini 316 KILSKGKHKIDGKSYYVTEDLYDVVPKDMAE-PYHLVVKDSQVHLDYPRKFITKNDGPPTVKVENPFIYESKSVVVSSWH 394
     S. cornubiensis 316 KILSKGKHKIDGKSYYVTEDLYDVVPKEMTE-PYHLVVKDSQVHLDYPRKFITKNDGPPTVKVENPFIYESKSVVVSSWH 394
      S. intermedius 316 KILSKGKHKIDGKSYYVTEDLYDVVPKDMSE-PYDFVVKDSQVHLDYPRKFITKNDGPPTVKVESPFIYESKSAVVSSWH 394
           S. lutrae 316 KVLSKGKHVIDDKKYYVTQDLYDVVPKSLKT-PYHFVVTDSQVHLEYPRQFITKNQGPPSVKVENPLIYESKSVVVSSWR 394
       S. schleiferi 318 KVLSKGKHKIDGKTYYVTKDLYDVVPKNLKK-PYHFIIDDNQVHLDYDRKFITKHDGPPKVEVENPFIYESKSVVISSWQ 396
        S. coagulans 316 KVLSKGKHKIDGKTYYVTKDLYDVVPKNLKK-PYHFVIDDNQVHLDYDRKFITKHDGPPKVEVENPFIYESKSVVISSWQ 394
       S. fleurettii 316 KVLSKGKHKIDGKTYYVTKDLYDVVPKNLKK-PYHFVIDDNHVHLDYDRKFITKHDGPPKVEVENPFIYESKSVVISSWQ 394
           S. hyicus 316 KILSKGEHKINGKTYYVTEDLYDVVPKSMTK-PYQFVVERGEVHLDYKRQFISKAYGPPKVEVEKPIVYQSKSFVVSSWK 394
          S. agnetis 316 KILSKGEHKINGKTYYVTQDLYDIVPKSMTK-PYQLVIENGEVHLNYKRQFISKAYGPPKVEVEKPIVYQSKSFVVSSWK 394
      S. chromogenes 316 KVLAKGEHKINGKAYYVTEDLYDVVPKGMTK-PYRFVIEKGEVHLEYDRQFISKAYGPPTVTVEKPIVHQSKSFVVSSWK 394
           S. aureus 319 NVLSKGTHKINGKTYYVKENLYDVVPKDMNQkDYKFIIKDGKVHLDYDRQFLTKDDGPPKVDVTKPLLHKANTIAQTTWK 398

S. pseudintermedius 395 AFPILTSLAFLVIVLILAFIL-RRLLKLLFRK-  425
         S. delphini 395 AFPVLTTLALLVIVLILAFIL-RRLLKLLFRK-  425
     S. cornubiensis 395 AFPVLTTLAFLVIVLILAFIL-RRLLKLLFRK-  425
      S. intermedius 395 AFPVLTTLASLVIVLVLALLL-RRLLKSLLRK-  425
           S. lutrae 395 AYPVLTTLAFAVIVLILAFIL-NRIFSLLRKK-  425
       S. schleiferi 397 THPWMTILAFGVIVVFLSIVF-YSLMSRFRRK-  427
        S. coagulans 395 THPWMTILAFGVIVVFLSIVF-YCLMSRFRRK-  425
       S. fleurettii 395 THPWMTILAFGVIVVFLSIVF-YSLMSRFRRK-  425
           S. hyicus 395 DHPLLTTLGFIFLIGFLSVIIyYMLFLTIKRR-  426
          S. agnetis 395 DHPILTSLGFIFLIGFLSVIIyYLLFLTIKRR-  426
      S. chromogenes 395 DHPILTSLGLLFLIGFLSVIIyYLLFLTFNRRk  427
           S. aureus 399 EHPIVTFIALALLAIACILIV-RSIIHLLFKRk  430
```
